# The role of hematological parameters in COVID-19 patients in the emergency room

**DOI:** 10.2217/bmm-2020-0317

**Published:** 2020-07-21

**Authors:** Eren Usul, İshak Şan, Burak Bekgöz, Ali Şahin

**Affiliations:** ^1^Sincan Dr Nafiz Körez State Hospital, Emergency Service, Ankara, Turkey; ^2^Department of Emergency Medicine, University of Health Sciences, Ankara City Hospital, Ankara, Turkey; ^3^Etimesgut State Hospital, Emergency Service, Ankara, Turkey

**Keywords:** biomarkers, COVID-19, diagnosis, emergency medicine, infection, neutrophil lymphocyte ratio, peripheral blood, platelet lymphocyte ratio, pneumonia, systemic inflammatory index

## Abstract

**Aim:** In this study, the roles of biomarkers from a peripheral blood sample in the diagnosis of Coronavirus Disease of 2019 (COVID-19) patients who have visited the emergency room have been evaluated. **Materials & methods:** Peripheral blood parameters, systemic inflammatory index (SII), neutrophil lymphocyte ratio (NLR) and platelet lymphocyte ratio were compared in patients with and without confirmed COVID-19 infection. **Results:** Comparisons made according to real-time PCR test results revealed that while no statistically significant difference was observed between test groups (negative-positive) regarding lymphocyte and platelet lymphocyte ratio values (p > 0.05), a statistically significant difference (p < 0.05) was found between the test groups regarding platelet, hemoglobin, leukocyte, neutrophil, NLR and SII values. **Conclusion:** Leukocyte, neutrophil, platelet count, NLR and SII values can be used in the diagnosis of COVID-19.

On 29 December 2019, pneumonia cases were detected in a hospital in Wuhan, China [1]. The Chinese Center for Disease Control and Prevention then confirmed, after studying throat cultures from patients, that these cases were caused by a new type of beta-coronavirus [2]. The exact mode of transmission of the disease is not known, and while the current information is limited, it supports person-to-person transmission. The most possible routes of transmission are thought to be droplet-based and contact-based [3].

With rapid spreading of the outbreak, the WHO announced a public health emergency of international concern [4]. Thus, the 2019-nCoV infection has led to a pandemic that has affected millions worldwide. In general, coronaviruses can cause various conditions, including respiratory, enteric, neurological and hepatic diseases [5]. Severe respiratory disease can be seen in the elderly and specific patient groups, such as those with underlying medical conditions [6]. Early diagnosis is vital when considering the short time of onset of acute respiratory distress syndrome after admission to hospital and the high mortality rates in the Coronavirus Disease of 2019 (COVID-19) [7].

Blood tests have an important role in early diagnosis of the disease, considering the information they provide to physicians regarding the inflammatory process. This information includes leukocyte count and characteristics such as neutrophil- or lymphocyte-dominance, inflammation (CRP), collateral organ damage (acute renal failure, acute liver failure) and the severity of the disease. Furthermore, biomarkers provide information regarding the nature of pneumonia, meaning that physicians can determine whether a disease is bacterial or due to other etiologies by analyzing blood test results [8].

Complete blood counts (CBC) are easily performed and inexpensive. Included in the CBC are values such as white blood count, neutrophil, lymphocyte and platelet count (PLT), mean platelet volume and certain ratios of these values. These can be used as inflammatory markers. Neutrophils are the most characteristic cell type among the white blood cells and are an important component of the immune system. Regulated by mast cells, epithelial cells and macrophages, neutrophils also take part in inflammatory processes. The role of lymphocytes in both inflammation and infections is evident. Additionally, thrombocytes also have importance in the regulation of various inflammatory processes. While these parameters may be used as inflammatory markers by themselves, their ratios to one another may also be indicators of early inflammation* [9–11]. Circulating leukocytes respond to stress by increasing neutrophils and reducing lymphocytes; the ratio of these two parameters is also used as an inflammatory marker [12].

Considering previous research, the use of circulating biomarkers representing inflammation and the immune system have been considered as a prognostic indicator in COVID-19-positive patients. However, their utility in terms of diagnosis has not been explored [13]. In this study, the roles of biomarkers from a peripheral blood sample in the diagnosis of COVID-19 patients who have gone to the emergency room (ER) of a second level state hospital have been examined.

Systemic inflammatory index (SII) = thrombocyte count × neutrophil count/lymphocyte count. Neutrophil lymphocyte ratio (NLR) = absolute neutrophil count/absolute lymphocyte count. Platelet lymphocyte ratio = absolute PLT/absolute lymphocyte count.

## Materials & methods

This retrospective study was carried out in line with research regulations, including the approval of the Ethics Committee of the University of Health Sciences, Dr Abdurrahman Yurtaslan Oncology Health Practice and Research Center dated 20 May 2020 and numbered 2020-05/609. This study also agrees with the principles of the Declaration of Helsinki of the World Medical Association.

In this study, 282 patients who have gone to the emergency department of Ankara’s Etimesgut State Hospital between 15 March 2020 and 15 April 2020 and who have been considered as possibly having COVID-19 were included. The recommended criteria established by the Scientific Committee of the Ministry of Health were used for the selection of possible COVID-19 patients. These criteria, consist of patients with either at least one sign or symptom of either fever or acute respiratory disease (cough and respiratory distress), or the presence of clinical features that are unexplainable by any other disease, or history of travel to another country in the previous 14 days before onset of symptoms of the patient or a relative, or close contact with a patient confirmed positive for COVID-19 by real-time PCR (RT-PCR) [14]. Patients from whom a throat swab was obtained and those who were thereafter hospitalized with an initial diagnosis of COVID-19 were studied retrospectively. Only patients above the age of 18 years were included in the study. The presenting complaints, epidemiological features and blood test results of patients were obtained from the patient files. Only the results of the initial RT-PCR and CBC tests were used and no further tests were performed on these patients.

The decision to obtain a CBC from incoming patients was made by the ER’s attending physician, and later, the blood sample was taken by the ER nurse. The blood sample was studied in the ER laboratory by the laboratory technician using the Horiba Medical Pentra DF Nexus analyzer. The CBC results obtained from this analysis were studied and approved by a biochemistry specialist.

The analysis of the data was done using the IBM SPSS 25.0 and Med Calc 15.8 statistical package programs. The normality of the data distribution was determined by the Shapiro–Wilk test, histogram and Q-Q plots. A Chi-square test was used to analyze the categorical variables of the patients, which were expressed as a number and percentage. For parametric continued variables, the independent samples t-test was used for analysis and they were presented as a mean and standard deviation. Nonparametric variables were analyzed using the Mann–Whitney U test and presented as the median and interquartile range. To define risk ratios, the Binary Logistic Regression test was used to assess the diagnostic utility of several biomarkers, including the SII. A receiver-operating characteristic (ROC) curve was generated, the Youden’s index (J) used for cut-off values in the diagnosis of COVID-19 and the area under the curve (AUC) was calculated. The 95% CI was calculated whenever appropriate, and a two-tailed p < 0.05 was considered statistically significant.

## Results

Of the 282 patients included in the study, 62.8% were male and the average age was 47.6 ± 16.9 years. RT-PCR analyses of 163 patients (57.8%) were positive. The most common complaints during ER visits were fever (37.2%), cough (15.2%), sore throat (5.3%), shortness of breath (3.9%) and myalgia (3.2%) (Table 1).

**Table 1.** Comparisons of patient features according to PCR test results.

Patient features		Total (n = 282)	Negative (n = 119)	Positive (n = 163)	p-value
Gender	Female	105 (37.2%)	55 (46.2%)	50 (30.7%)	**0.008**^‡^
	Male	177 (62.8%)	64 (53.8%)	113 (69.3%)	
Age (years)^†^	General	47.6 ± 16.9 46 (18–91)	49.6 ± 18.5 49 (33–64)	46.2 ± 15.5 43 (34–58)	0.097^§^
	Female	53.6 ± 17.7 56 (18–91)	54.1 ± 19.8 57 (36–68)	53.1 ± 15.3 56 (41–61)	0.780^§^
	Male	44.1 ± 15.4 41 (19–83)	45.7 ± 16.5 45 (31–57)	43.1 ± 14.7 40 (31–55)	0.286^§^
Symptoms	Fever	105 (37.2%)	70 (58.8%)	35 (21.5%)	
	Cough	43 (15.2%)	15 (12.6%)	28 (17.2%)	
	Fever – sore throat	15 (5.3%)	3 (2.5%)	12 (7.4%)	
	Dyspnea	11 (3.9%)	7 (5.9%)	4 (2.5%)	
	Myalgia	9 (3.2%)	6 (5.0%)	3 (1.8%)	
	Malaise and fatigue	3 (1.1%)	1 (0.8%)	2 (1.2%)	
	Fever – shortness of breath	2 (0.7%)	1 (0.8%)	1 (0.6%)	
	Headache	2 (0.7%)	2 (1.7%)	–	
	Chest pain	2 (0.7%)	2 (1.7%)	–	
	Rhinorrhea	1 (0.4%)	1 (0.8%)	–	

† Mean ± standard deviation/median (interquartile range: Q1–Q3).

‡ Chi-Square test.

§ Independent samples t-test.

The value in bold font has a statistically significant difference.

Comparisons made according to the PCR test results of patients included in the study, revealed that no difference of statistical significance regarding age and test results (negative or positive) was found (p > 0.05). Binary logistic regression was used to further evaluate whether age was a risk factor and as a result, it was determined that age of the patients was not a risk factor for COVID-19 (p = 0.098; OR: 0.998; 95% CI: 0.974–1.002). Furthermore, a significant difference was observed regarding gender and test results (p < 0.05). A positive test result among males was more commonly observed than in females. Also, those with a negative test result were more commonly symptomatic than those with a positive test result (Table 1).

Comparisons made according to the RT-PCR test results revealed that while no statistically significant difference was observed between test result groups (negative or positive) regarding lymphocyte and platelet lymphocyte ratio values (p > 0.05), a statistically significant difference (p < 0.05) was found between the test result groups regarding platelet, hemoglobin, leukocyte, neutrophil, NLR and SII values. In patients with negative test results, it was found that platelet, leukocyte, neutrophil, NLR and SII values were higher, whereas hemoglobin was found to be higher in patients with positive test results. Also, hemoglobin was found to be higher in male patients who tested positive for COVID-19 (Table 2).

**Table 2.** Comparisons of hematological parameters according to PCR test results.

Hematological parameters	Negative^†^ (n = 119)	Positive^†^ (n = 163)	p-value
Platelets	233.0 ± 72.3 226.0 (185.0–275.0)	210.0 ± 72.3 198.0 (168.0–241.0)	**0.009**^‡^
Hemoglobin	13.9 ± 1.8 14.0 (12.5–15.4)	14.7 ± 1.7 14.8 (13.3–15.9)	**0.000**^‡^
Leukocytes	9.2 ± 3.7 8.4 (6.9–10.6)	6.0 ± 3.9 5.8 (4.5–7.0)	**0.000**^‡^
Neutrophils	6.2 ± 3.5 5.5 (4.1–7.6)	3.5 ± 2.0 3.1 (2.4–4.1)	**0.000**^§^
Lymphocytes	2.2 ± 2.5 1.9 (1.4–2.4)	2.8 ± 5.5 1.8 (1.4–2.3)	0.223^‡^
NLR	3.8 ± 3.2 2.8 (1.9–5.0)	2.0 ± 1.5 1.7 (1.2–2.4)	**0.000**^§^
PLR	133.5 ± 59.3 121.3 (91.2–164.1)	119.8 ± 58.5 113.3 (82.2–147.5)	0.081^§^
SII	864.4 ± 718.0 659.8 (409.3–979.1)	427.5 ± 347.0 345.9 (218.5–501.2)	**0.000**^§^

† Mean ± standard deviation/median (interquartile range: Q1–Q3).

‡ Independent samples t-test.

§ Mann–Whitney U test.

The values in bold font have a statistically significant difference.

NLR: Neutrophil lymphocyte ratio; PLR: Platelet lymphocyte ratio; SII: Systemic inflammatory index.

**Figure 1.**
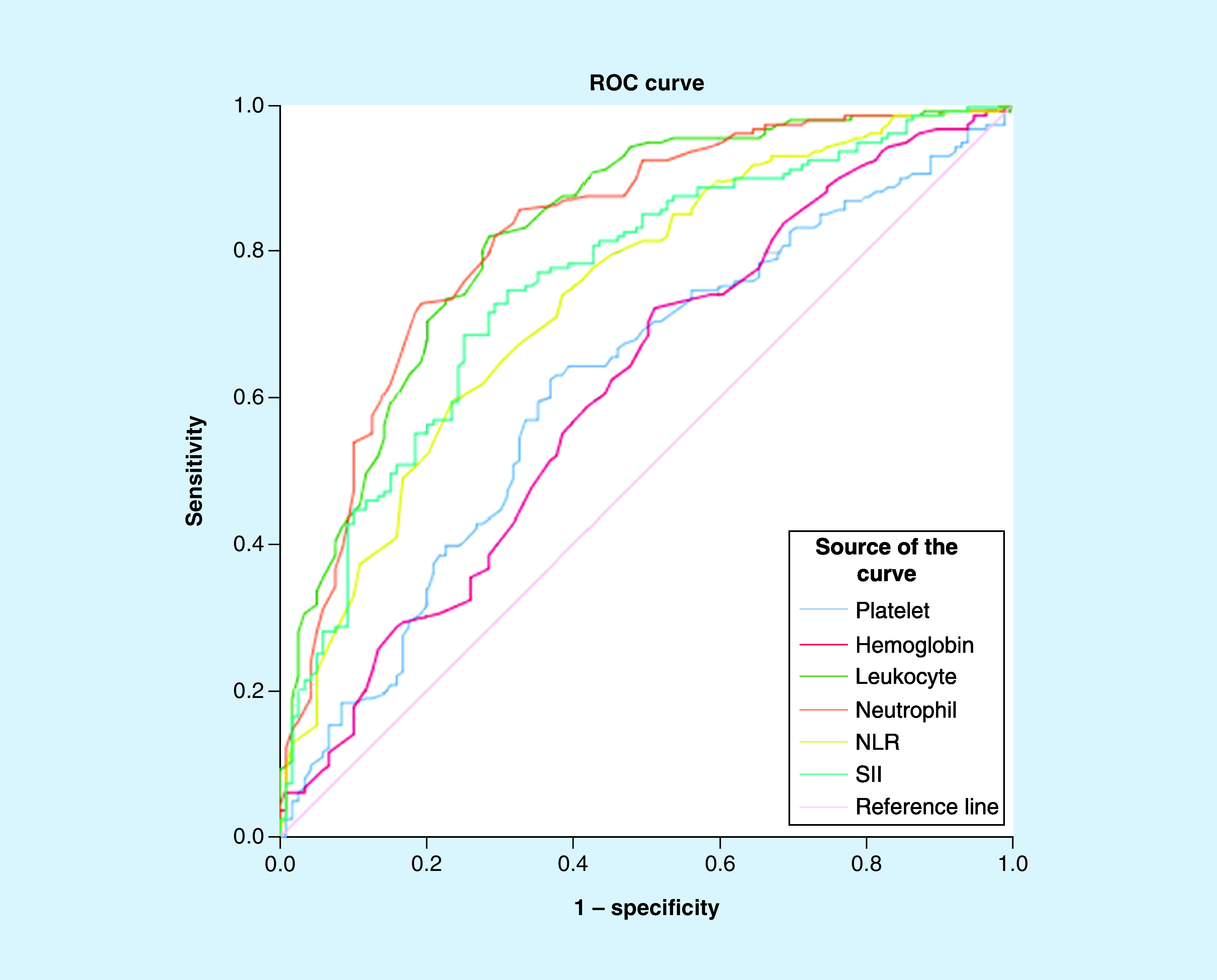
Receiver-operating characteristic curve for significant markers in the prediction of Coronavirus Disease of 2019 positive patients. NLR: Neutrophil lymphocyte ratio; ROC: Receiver-operating characteristic; SII: Systemic inflammatory index.

Since there was a statistically significant difference between negative and positive groups according to parameters (platelet, hemoglobin, leukocyte, neutrophil, NLR and SII), they were then studied with ROC analyses. Regarding the observations made by ROC analyses, the following information concerning patients with COVID-19 diagnoses were found (Table 3 & Figure 1): The cut-off point for platelet values was found to be ≤211 (AUC = 0.618; p = 0.001; 95% CI: 0.558–0.0675; J = 0.256). The cut-off point for hemoglobin was found to be >13.6 (AUC = 0.612; p = 0.001; 95% CI: 0.553–0.670; J = 0.211). The cut-off point for leukocyte values was found to be ≤7.2 (AUC = 0.828; p < 0.001; 95% CI: 0.779–0.871; J = 0.536). The cut-off point for neutrophil values was found to be ≤3.9 (AUC = 0.826; p < 0.001; 95% CI: 0.776–0.868; J = 0.537). The cut-off point for NLR was found to be ≤1.8 (AUC = 0.739; p < 0.001; 95% CI: 0.684–0.790; J = 0.360). The cut-off point for SII values was found to be ≤479.1 (AUC = 0.760; p < 0.001; 95% CI: 0.706–0.808; J = 0.437).

**Table 3.** Recommended cut-off values for significant markers in the prediction of Coronavirus Disease of 2019 (+) patients.

Markers	AUC	Cut off	Sensitivity	Specificity	95% CI	p-value^†^
Platelets	0.618	≤211	62.6	63.0	0.558–0.675	**0.001**
Hemoglobin	0.612	>13.6	72.4	48.7	0.553–0.670	**0.001**
Leukocytes	0.828	≤7.2	82.2	71.4	0.779–0.871	**0.000**
Neutrophils	0.826	≤3.9	73.0	80.7	0.776–0.868	**0.000**
NLR	0.739	≤1.8	59.5	76.5	0.684–0.790	**0.000**
SII	0.760	≤479.1	74.9	68.9	0.706–0.808	**0.000**

† ROC curve.

The values in bold font have a statistically significant difference.

AUC: Area under the curve; NLR: Neutrophil lymphocyte ratio; ROC: Receiver-operating characteristic; SII: Systemic inflammatory index.

Additional analysis of these parameters was performed in order to find the distribution of patients among negative and positive groups according to their cut-off values. Comparisons made between these new groups that were formed according to the cut-off values, revealed that a statistically significant difference existed between the groups for these parameters (Table 4).

**Table 4.** Comparison of results according to cut-off points.

Hematological parameters		Negative (n = 119)	Positive (n = 163)	p-value
Platelets	>211	75 (55.1%)	61 (44.9%)	0.000
	≤211	44 (30.1%)	102 (69.9%)	
Hemoglobin	≤13.6	58 (56.3%)	45 (43.7%)	0.000
	>13.6	61 (34.1%)	118 (65.9%)	
Leukocytes	>7.2	85 (74.6%)	29 (25.4%)	0.000
	≤7.2	34 (20.2%)	134 (79.8%)	
Neutrophils	>3.9	96 (68.6%)	44 (31.4%)	0.000
	≤3.9	23 (16.2%)	119 (83.8%)	
NLR	>1.8	94 (56.6%)	72 (43.4%)	0.000
	≤1.8	25 (21.6%)	91 (78.4%)	
SII	>479.1	82 (66.7%)	41 (33.3%)	0.000
	≤479.1	37 (23.3%)	122 (76.7%)	

NLR: Neutrophil lymphocyte ratio; SII: Systemic inflammatory index.

## Discussion

Considering the high infectivity and mortality rates of COVID-19, early diagnosis of the disease is essential. The definitive diagnosis of this disease is made by proving a viral presence in real-time PCR analyses. Due to factors such as the high number of samples, limited number of staff trained in performing the aforementioned tests and insufficient lab capacities, the time it takes to receive results can be prolonged. Therefore, every parameter allowing for early diagnosis is vital. In this study, the possibility of diagnosing COVID-19 early in ER visits by a simple, inexpensive, easily accessible test, such as a CBC, has been examined.

Nasopharyngeal swabs were obtained from 282 suspected cases and of these, 163 patients were confirmed positive for COVID-19 by RT-PCR. Out of all the patients diagnosed with COVID-19, 69.3% were males and the average age was 46.2 ± 15.5 years. The average age was found to be lower in males compared with females. In a study conducted by Guan *et al.*, the median age was 47 and 52.1% of the patients were male [15]. Another study by Li *et al.* revealed that 56% of all patients were male and the median age was 59 [16]. Furthermore, another study conducted by Xu *et al.* showed a median age of 41 and 56% of the patients were male [17]. Thus, it can be said that COVID-19 is seen more frequently in males and in middle-aged patients.

Upon evaluation of common complaints during ER visits, fever, cough and a sore throat were the most observed, followed less often by myalgia, malaise and fatigue. The results of this study were found to be noticeably compatible with that of previous research. The research conducted by Yang *et al.* also revealed fever and cough to be the most common complaints [18]. In a study by Guan *et al.*, fever and cough and less frequently nausea, vomiting and diarrhea, were observed [15]. The study by Huang *et al.* showed that fever (40/41 patients [98%]), cough (31/41 patients [76%]) and myalgia or fatigue (18/41 patients [44%]) were the most commonly seen symptoms at onset of the disease [7].

In the results of this study, which are also consistent with previous research, low thrombocyte, leukocyte and neutrophil counts were revealed in COVID-19 positive patients. Thus, it can be said that thrombocytopenia, leukopenia and neutropenia may be indicative of COVID-19 disease. Likewise, thrombocytopenia and leukopenia were noted in Guan *et al.*’s study [15]. The thrombocyte count was also found to be low in the study by Assiri *et al.* [19] and leukopenia was noted in another study conducted by Xu *et al.* [17]. In general, while the leukocyte count was lower than 10,000 in viral pneumonias, leukocytosis was seen in bacterial pneumonias with a leukocyte count of more than 50,000 [20]. Additionally, Xu *et al.* revealed in their study that thrombocyte counts are significantly low in pneumonia patients and that this decrease is directly proportional to the patients' clinical status [21]. In a study by Fan *et al.* mild thrombocytopenia and leukopenia was observed in some patients at first admission who were COVID-19 positive [22]. The effects of viral pneumonia on the immune system show a decrease in thrombocyte, leukocyte and neutrophil counts.

Also, hemoglobin levels in COVID-19 positive patients were found to be significantly higher than in COVID-19-negative patients. While no significant difference was observed among females regarding hemoglobin, higher hemoglobin levels were seen in COVID-19 positive male patients. It is possible that these results are also affected by other reasons, such as the presence of comorbidities or anemia, and habits such as cigarette smoking. The patient files used for this study did not include a detailed patient history, and thus, their effect on hemoglobin levels were not accounted for. Also, the normal hemoglobin level in the female population is lower than that of males [23]. Since around 70% of the positive patient group is comprised of males in this study, this is likely to also have an effect on the results.

An apparent relationship, although not certainly proven, exists between a bacterial infection and neutrophilia, and a viral infection and lymphocytosis. Accordingly, NLR of peripheral blood has been used to distinguish between these types of infections [24,25]. In a retrospective study concerning hospitalized patients with a fever of an unknown origin, it has been shown that NLR is higher in those with fever due to bacterial infections than those with fever due to a viral etiology [26]. In the study by Zhang *et al.*, NLRs were used as an early diagnostic marker for aiv-H7N9 patients [27]. Ai-Ping Yang *et al.* found an AUC of 0.743, with a cut-off of 3.3, specificity of 0.636, and a sensitivity of 0.88 for NLR in determining the prognosis for seriously ill COVID-19 patients [18]. Moreover, in the study conducted by Sun *et al.*, an AUC of 0.88 for NLR was found for serious COVID-19 positive patients at their first visit to the ER [28]. Similarly, this study found a significantly lower NLR at first visit to the ER for patients with positive test results. Low NLR levels can therefore possibly be used as a diagnostic marker for COVID-19. The following has been observed for NLR: AUC = 0.739, cut-off = 1.8, specificity = 0.765 and sensitivity = 0.595.

A recently proposed prognostic score is the SII, which relies on thrombocytes, neutrophils and lymphocytes. As an index defining the instability in the inflammatory response, the SII has been proposed as a prognostic indicator in the follow-up of sepsis patients [29]. In addition, SII has been found to be useful in predicting the prognosis of small cell lung cancer and hepatocellular carcinoma [30,31]. In this study, SII was found to be significantly low for COVID-19-positive patients, meaning that it can also be used while diagnosing COVID-19. The cut-off value for SII was noted as being ≤479.1, and the AUC = 0.760 with a sensitivity of 74.9%, and a specificity of 68.9%.

In conclusion, the results demonstrate that low values for NLR, SII, absolute neutrophil and lymphocyte counts, and PLTs may have diagnostic properties concerning COVID-19. The widest area under the ROC curve was of absolute leukocyte and neutrophil counts, followed by the AUC’s of SII and NLR.

## Limitations

The greatest limitation of this study was the inadequate number of patients. Studies conducted with a larger patient group will better portray the importance of biomarkers from peripheral blood tests in the diagnosis of COVID-19 patients. Another limitation was the fact that patients who deteriorated, meaning those who had increased respiratory distress possibly requiring invasive ventilation, advanced life support or intensive care, were transferred to an education and research hospital, thus, the follow-ups and prognoses of these patients remain unknown. Furthermore, it was not possible to include false-negative results since secondary testing with RT-PCR was not performed. Also, patient histories including comorbidities or personal habits (e.g., cigarette smoking and alcohol use) were not sufficiently obtained from the patient files, meaning their effects on the results were not accounted for.

## Conclusion

The definitive diagnosis of COVID-19 was made by RT-PCR analysis, but this was a time-consuming and less accessible test. With this test, the time it takes to diagnose and treat patients can be delayed. In our study, low values of leukocytes, neutrophils, platelets and high values of hemoglobin found with a CBC test which is easily available in ERs were found to be valuable in terms of the initial diagnosis of COVID-19. In addition, low values of NLR and SII were also indicative of COVID-19.

**Summary points**

- A statistically significant difference (p < 0.05) was found between this test result groups regarding platelet, leukocyte and neutrophils.
- A statistically significant difference (p < 0.05) was found between this test result groups regarding systemic inflammatory index (SII) and neutrophil lymphocyte ratio (NLR).
- Hemoglobin was found to be higher in male patients who tested positive for Coronavirus Disease of 2019.
- No statistically significant difference (p < 0.05) was found between platelet lymphocyte ratio and lymphocyte ratios.
- Receiver-operating characteristic analysis was performed to determine the NLR, SII, neutrophil, platelet, hemoglobin and leukocyte values. The area under the curve area was the highest in leukocyte, followed by neutrophil, SII and NLR.
